# A System of Medicine

**Published:** 1899-12

**Authors:** 


**A System of Medicine.** By Many Writers. Edited by THOMAS CLIFFORD ALLBUTT, M.D., F.R.S. Vol. VI. Pp. x., 944. London: Macmillan and Co., Limited. 1899.

Nearly half of this volume deals with the diseases of the circulatory system, continued from the fifth. Dr. G. Newton Pitt gives a very complete account of the right-sided valvular diseases, and Sir R. Douglas Powell of angina pectoris. His well-known division of angina into the two varieties, the vaso-motor and the graver form, including both the secondary and the primary cardiac angina, is now usually accepted as authoritative. Dr. Frederick T. Roberts follows with nearly a hundred pages on diseases of the mediastinum and thymus gland, with intrathoracic growths and tumours.

The article on Thrombosis by Professor Welch, of Baltimore, occupies some seventy pages, and is quite a noteworthy addition to the literature of the subject; that on Embolism is a little shorter, but is also a complete and very comprehensive monograph; it is of interest that "in the great majority of cases, fat-embolism is entirely innocuous . . . it is only in the comparatively rare instances of extensive fat-embolism that effects of any consequence are produced" (pp. 258-9). A short article on Phlebitis is by Mr. H. H. Clutton; and that on Arterial Degenerations and Diseases is by Dr. F. W. Mott. The veteran Professor Sir W. T. Gairdner writes an article of ninety pages on Aneurysm of the Aorta, in which he states that

"we have in iodide of potassium a remedy which ought to be carefully and deliberately employed in most, if not in all, cases of aneurysm of the aorta." A short account, by Dr. H. D. Rolleston, of Aneurysms of Arteries in the Abdomen and of Diseases of the Lymphatic Vessels concludes this portion of the volume.

Four chapters on Diseases of the Muscles include myositis, myotonia congenita, idiopathic muscular atrophy and hypertrophy, and facial hemi-atrophy and hemihypertrophy. Then follows a chapter on the General Pathology of the Nervous System by Dr. W. Bevan Lewis, giving a succinct description of the modern conception of the neuron and the neuronic system, and pointing out "that the morphological unity of the neuron and the doctrine of contiguity rather than continuity of related neurons, favour a strict limitation of degenerative changes, arising in a nerve chain, to the individual neuron affected." Dr. Sherrington describes Tremor, "Tendon - Phenomenon," and Spasm, of which the physiological basis "is a compound reaction from two integrated tissues of the body—the nervous and the muscular." These two factors are considered in detail as a preliminary to the clinical and pathological section by Dr. Seymour J. Sharkey. Chapters follow on Trophoneuroses, including the Neurotrophic Affections of Bones and Joints; the Neurotrophic Diseases of Soft Tissues; Adiposis Dolorosa; Raynaud's Disease; and Erythromelalgia. These chapters are very complete, and introduce a great variety of interesting topics relating to the pathology of many obscure affections.

Further chapters on diseases of the nervous system and of the spine conclude the sixth volume of a work which well repays careful study, and which is rapidly approaching completion.

